# An empirical examination of customer advocacy influenced by engagement behaviour and predispositions of FinTech customers in India

**DOI:** 10.12688/f1000research.74928.2

**Published:** 2022-05-23

**Authors:** Archana Nayak Kini, Savitha Basri

**Affiliations:** 1 Manipal Institute of Management, Manipal Academy of Higher Education, Manipal, Karnataka, 576104, India

**Keywords:** customer engagement behaviour, customer advocacy, e-word-of-mouth, emotions, perceived benefits, self-concept, FinTech, technology enabled services

## Abstract

**Background:** The extensive adoption and usage of emerging technologies furthered by the global coronavirus disease 2019 (COVID-19) pandemic, has reduced direct face to face communications. New FinTech (financial technology) apps and technologies are flooding the Indian digital payments market and competitors are striving hard to attract and retain their customers. Especially when customer engagement behaviours (CEBs) are digital in nature, it is essential to gauge the intrinsically driven customer motivations which drive a positive CEB. The objective of this paper was to empirically test the influence of customer-based antecedents such as emotions, moral identity, self-concept, communal focus, perceived cost and perceived benefits on non-transactional experiential customer engagement behaviours (CEBs) and CEB’s effect on customer advocacy in the FinTech industry.

**Methods:** Data from 380 financial app users in south India were gathered by administering a survey that captured customer predispositions, CEBs, and customer advocacy. Structural equation modelling (SEM) using smart PLS (partial least squares) 3.0 was applied to test the theoretical model.

**Results:** Results indicate that CEB fully mediates the relationship between self-concept and customer advocacy. The positive CEBs get formed through customer predispositions leading to referral/advocacy behaviours.

**Conclusions:** This paper provides directions for FinTech practitioners, marketers, technologists, and academicians to devise marketing strategies customized to customer needs and factors. This is one of the first research studies to demonstrate and empirically validate the CEB model for the FinTech industry during the COVID-19 pandemic.

## Introduction

Since the liberalization of the financial services industry in India in the 1990s and the global financial crisis in 2008, the financial sector has undergone a series of changes and reforms. In India, the passage of the information technology (IT) Act of 2000 provided much-needed momentum to financial technology (FinTech) operations by establishing legal validity. With increased internet usage, the reduction of entry hurdles, and the easing of regulations, the post-crisis period from 2010 saw a tremendous rise in the use of financial technology ( Feyen *et al*., 2021). FinTech firms provide financial services on digital platforms by combining innovative business models and advanced technologies such as artificial intelligence (AI), social media, data analytics and robotics acting as enablers. FinTech is setting a new paradigm in the design and delivery of financial products and services through alternative channels ( Lee & Shin, 2018). The key service offerings emerging on digital platforms in India include peer-to-peer (P2P) lending services such as Lendbox, Shiksha Financial, payment services such as Google pay, PhonePe, Paytm, BHIM, personal advisory services such as FundsIndia.com, Scripbox, PolicyBazaar, and BankBazaar, among others ( Cyrill, 2018). India's digital payment is predicted to comprise 2.2% of the world's payments which is poised to reach $12.4 trillion in another 3 to 5 years ( Payments Council of India and PWC Report, 2020). The coronavirus disease 2019 (COVID-19) pandemic has ushered in a new era of contactless mobile banking. Retail payments in India through various digital channels rose from $37.7 billion in financial year (FY) 2018-19 to $48.35 billion in FY 2020-21 (1 US$ = Rs 74.25, 03 August 2021) and mobile phone usage for banking operations in India grew from 61% in 2017 to 87% in 2021 ( RBI, 2021). India along with China accounts for the highest FinTech adoption rate of 87% compared to the global adoption rate which is at 64% ( Ernst & Young, 2017). An increase in the adoption of FinTech solutions was due to the COVID-19 pandemic. In emerging and developing markets such as India, FinTech has helped expand access to affordable financial services to the common masses in times of lockdown and social distancing ( RBI, 2021).

FinTech is prompting drastic changes in the way financial transactions - especially payments and advisory services - are perceived, marketed and consumed ( Mention, 2021). The most significant result of FinTech is that it has shifted the focus on customer's convenience and needs with anytime, anywhere banking, breaking down the time and distance barriers that traditional banking imposes. Consumers are also finding it easier to browse around and switch service providers in search of more product options, greater features, lower prices, or discounts. Customers, on the other hand, are perplexed because the market is swamped with services and products from a variety of providers. Additionally, unlike traditional banking techniques, FinTech is perceived as sophisticated and thus difficult to use by older cohorts who are not tech-savvy. As a result, it has become incredibly challenging for FinTech firms to manage consumer demands, establish or retain a customer base, and hence obtain a competitive advantage.

Can gaining a certain level of customer advocacy help FinTech firms beat the competition in this emerging and dynamic market? Can customers be motivated to interact positively with other customers by nurturing their self-concept, emotional states of mind and pre-dispositions? Also, can achieving a certain level of engagement with the FinTech brand ensure customers endorse the products and services and become its advocates? It has been observed over the years that a services firm can gain a competitive advantage by retaining, sustaining and nurturing its customer base ( Anderson *et al*., 2004) by looking beyond the repurchase behaviour. Digital and social media with their richer media not only help firms to connect but also communicate information and emotions to their immediate customers and also between customers ( Sashi, 2012). Hence customer-to-customer (C2C) interactions help companies promote brand advocacy by listening and learning about customers’ needs, influencing them through social media ‘thought leaders’ and also considering customer opinions ( Constantinides & Fountain, 2008).

Customization, personalization efforts, and systems that engage with customers are known to affect favourable e-WOM(e-word of mouth), reviews and testimonials. To personalise, it is useful to know consumers' channel preferences, breadth of engagement, time, effort, and money spent, as well as the kind of activities they engage in. These can then predict the intensity of their referrals and product reuse ( van Doorn *et al.*, 2010). In a COVID-19 world, where all these engagement behaviours are digital, interactive, brand centred and non-transactional, it becomes essential to gauge the intrinsically driven customer motivations. Customers’ helping behaviour where they guide other customers with useful suggestions or solve a problem, or customers’ sense of community could aid them in engaging with the brand. The emotions of happiness, anger, contentment, or disgust can motivate a desirable or an undesirable customer engagement behaviour (CEB) or a referral, working as a differentiator amidst competition. Establishing emotional connections with customers can build trust and in turn assist these firms to face several challenges and intense competition in the FinTech industry.

The goal of this quantitative study is to empirically test the conceptual model on the influence of customer-based predispositions on non-transactional CEBs, and its strength in predicting the resultant advocacy behaviours in the emerging FinTech industry especially in covid pandemic times. The study sample were FinTech app customers who use Google Pay, PhonePe and so on for payment services and BankBazaar, PolicyBazaar for advisory services. The results of this study may guide marketing managers to formulate robust segmentation, positional strategies in line with the emotional, self-concept and individual needs so that they engage positively with the brand. Customer engagement and advocacy behaviours gained from these campaigns can help the companies to build and retain the competitive edge in the market.

## Literature review

Service-Dominant (SD) logic in the light of relationship marketing theory attempts to know the significance of customer-to-customer (C2C) relational and referral behaviours in developing and maintaining loyalty and advocacy. The customer engagement framework in this study endorses Oliver's (1999) theory elaborating the belief-attitude-behaviour formation. In addition, social exchange theory (SET) proposes that customers display positive thoughts, feelings or behaviours towards a product or brand in reciprocation to specific benefits from the brand ( Blau, 1964). CEBs arising through these processes help in spreading positive word-of-mouth ( Oliver, 1999), thus advancing the underlying theories and also inspiring a future research agenda in this direction.

In a technology-intensive financial services sector, the use of new information and technologies has led to a reduction in direct physical contact with the customer. The online customer communities facilitate customers to have an open dialogue to understand and solve their problems mutually, thus effectively engaging with the brand community. This has changed the traditional roles of a buyer and a seller in such exchange relationships. Thus, customers create and share content and thus intangibly advocate the product and service value to other customers, influencing their purchase decisions. Customer advocacy is a natural side-effect of the forces that create a strong service provider-customer relationship and is a dominant pointer of repeat buying ( Fullerton, 2011). An investment in the brand from an experiential approach consisting of CEBs determines customer advocacy ( van Doorn *et al*., 2010; Moliner *et al.,* 2018).

**Figure 1.**
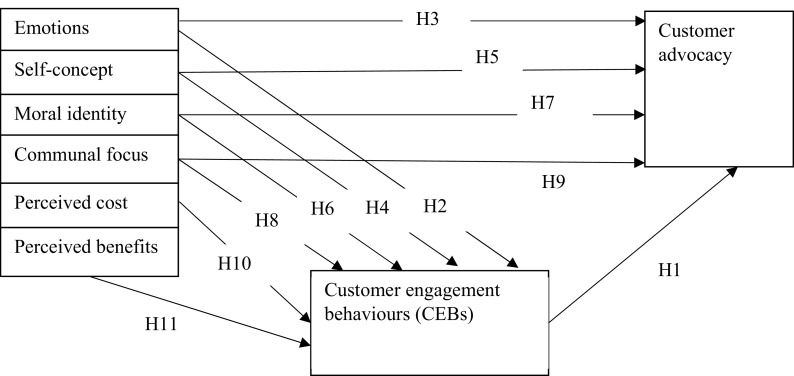
Theoretical model.

CEB denotes firm or brand centred behaviours that go beyond transactions but have positive or negative manifestations depending on the motivational drivers of the customer and are measured by valence (positive or negative), form/modality, scope (temporal and geographic), choice of channel and customer goals ( van Doorn *et al*., 2010). The form/modality component depicts ways in which customers express their engagement based on time and money resources available at their disposal. The scope encompasses customers’ extra-role behaviours such as providing product-related feedback helping the company improve the product or develop new products ( van Doorn *et al*., 2010; Kumar & Pansari, 2016). Customers' social media influence (CSMI) embodies C2C conversations over social media channels sharing their views on product benefits, thus influencing other customers of the brand community positively ( Kumar & Pansari, 2016). Customers' choice of the channel such as communication via phone, in-person, in a retail setting or via the internet (email/website) can impact the CEB in terms of immediacy, intensity, length and breadth of CEB ( van Doorn *et al*., 2010). CEBs are those non-purchase C2C behaviours where the customers influence the development of the firm's offering, brand identity through knowledge, skills, opinions, recommendations, online reviews and referrals on their own accord making it a part of experiential marketing ( Jaakkola & Alexander, 2014). Studies show that customer dialogue and engagement mediates the relation between emotionally connected satisfied customers and advocacy behaviours ( Moliner-Tina *et al*., 2019). As per Figure 1, CEB is a mediator and it is primarily motivated by customers’ pre-dispositions and which can predict customer behaviour defined by referrals and advocacy.

H1: Customer engagement behaviour positively impacts customer advocacy.

Customer predispositions are the customer-based factors, mainly the individual traits and predispositions of customers which influence the product, brand decisions and CEBs ( van Doorn *et al*., 2010). Levy and Hino (2016) and Bhat and Darzi (2016) substantiated that emotional and relational elements influence a bank's relation with customers rather than a purely transactional approach leading to bank advocacy. It was discovered that emotional states such as anger, regret, disgust towards the company or the brand can result in CEBs and customers' accumulated emotional experiences turning into actions and behaviours ( Bagozzi *et al*., 1999; Martin *et al*., 2008) and positive emotions generate positive CEBs and vice versa. Emotional connections with customers help in exhibiting the desirable CEB working as a differentiator amidst competition ( Garg *et al*., 2005; Levy & Hino, 2016). A quantitative study observed that the emotional dimension of engagement has a positive effect on advocacy intention as well as the intention to reuse mobile payment services compared to the cognitive dimension of engagement which only influences advocacy intention ( Glavee-Geo *et al*., 2019).

H2: Emotions have a positive effect on CEB.

H3: Emotions have a positive influence on customer advocacy.

Self-concept is defined as a “desire for positive recognition by others”. Moral identity is similar to self-identity and manifests in helping behaviour. The greater the moral identity of a customer, the more is the engagement behaviour with the brand and more likely is the helping behaviour. Customers who displayed a higher value for self-concept tend to warn other customers from negative product experiences or recommend installing the right application thus exhibiting a high degree of CEBs ( Hennig-Thurau *et al*. 2004). Similarly, customers with helping behaviour tend to participate in blogging or co-promote the brand with which they are involved ( Sundaram *et al*. 1998). Customers who are emotionally linked to brands symbolizing their self-concept are more inclined to champion the brand and disseminate favourable information about the product. They are thus less likely to be lured by competitors, are less difficult to persuade to stay, and are more eager to recommend the product ( Grisaffe & Nguyen, 2011; Jayasimha & Billore, 2016).

H4: Self-concept impacts CEB positively.

H5: Self-concept impacts customer advocacy positively.

H6: Moral Identity influences CEB in a positive manner.

H7: Moral Identity inspires customer advocacy in a positive manner.

Communal focus is the behaviour where the people belonging to a particular community are likely to voice their support for their community during difficult times ( He *et al.*, 2008). It was revealed that a strong agentic focus is likely to be displayed by the female gender while they voice their negative WOM or advocacy. The motivations for involving in positive e-WOM may vary from the impulses which drive a negative e-WOM as evident in communal focus ( van Doorn *et al*., 2010).

H8: Presence of communal focus is positively associated with CEB.

H9: Customer advocacy responds positively to moral identity.

Perceived cost and benefits are the perception by the customer related to the time, effort and money involved in buying and recommending the brand or product vis-à-vis the returns sought. Based on the perceived benefits and cost of engagement, the customer may engage in specific behaviours such as blogging or participation in online discussion forums or making donations to brand-related charity which has been defined as the form and modality of engagement ( van Doorn *et al*., 2010).

H10: CEB is positively influenced by perceived cost.

H11: CEB is positively motivated by perceived benefits.

## Methods

### Ethical considerations

Written informed consent was obtained from all survey participants before administering the survey questionnaire. The content of the form started with the information sheet introducing the research objectives, its outcomes and implications before getting respondents’ informed written consent. It contains statements on confirming that the data collected would be used for research and publication purposes keeping any personal details confidential (see *Extended data,* ( Nayak Kini & Basri, 2021c)). In addition, approval from the Manipal Institute of Management ethics committee was received for this empirical study in the Indian Fintech industry.

### Research design

Positivism philosophy was adopted for this study which helps to inquire the customers' emotions, attitudes and actions related to their app engagement scientifically and objectively. A cross-section of app users were initially chosen for the survey using a convenience sampling method and later this initial sample of app users helped to snowball their contacts who became the next set of respondents. The prevailing theories of engagement and exchange acted as a basis to understand the different phenomenon assisting in deductively formulating the various hypotheses to be tested for the theoretical model.

### Measurement tools/scales

The survey questionnaire used a 5-point Likert scale adapted from several researchers such as Levy and Hino (2016) to capture customers’ overall perceptions about self-concept having 5 items, van Doorn *et al.* (2010) for communal focus having 3 items, Hennig-Thurau *et al*. (2004) for moral identity having 4 items, Hayashi and Bradford (2014) for perceived cost and Moliner *et al*. (2018), Kumar and Pansari (2016) having 3 items, for CEBs having 18 items, and Moliner *et al*. (2018) and Han *et al*. (2008) for advocacy (having 5 items) concerning the specific apps they were using. For emotions, a 7-point Likert scale was used which had 4 items and was adapted from Wong (2004). The survey can be found as *Extended data* ( Nayak Kini & Basri, 2021b).

### Data collection

The universe for the study sample comprised of the customers of major FinTech payment and advisory applications such as Google Pay, PhonePe, Amazon Pay, BHIM, PayTM, BankBazaar, Policy Bazaar, and others in Karnataka located in southern region of South India. A pilot pretest with 38 respondents was conducted before administering the survey to a larger sample size during November 2020 and March 2021. A pilot pre-test is a trial run conducted before the final run for administering the survey. The pilot pretest conducted helps to identify any problems which might be encountered during the administration of the survey for data collection and find possible solutions before proceeding for the final study. The pre-test tried to gauge certain research design issues such as whether the respondents clearly understood the wording of the questions and also the time taken by the respondent to answer the whole questionnaire. The size of the sample for the pilot study was taken to be 10% of the sample size for the final study with sample size calculated to be 380 (formula below). The participants of the pilot test were chosen based on convenience sampling strategy but the initial criteria were that they had to belong to a city in Karnataka and also be using any of the above financial apps. We found out from the study that few questions and wording were hard for the respondents to understand, hence this helped to simplify the wording of few questions for the final study.

We had an initial contact list of participants chosen in the same way as that of the pilot study. These respondents were sent an unsolicited email introducing them to the survey and asking for their written consent. Once they provided the consent, the online survey link was shared with them and at the end of the survey, they were also asked to refer their contacts who used any of the FinTech apps. Thus, they helped refer their contacts’ email ids as well as the phone numbers. This new group of people referred more people from their network with a snowball effect. We were able to reach out to 450 participants who consented to fill out the survey through this process. This process was iterated until the final targeted sample size of 420 completed the survey. The response rate of the survey was observed to be at 93% ({420/450}*100).

For completed surveys, response bias occurs when individuals respond to a survey leading to misrepresentation of their true value due to reasons such as social desirability and a set pattern of questions while filling. Response bias can also develop when the researcher's intent and the respondent's comprehension are not in sync. By using reversely coded, neutrally phrased, and brief questions, as well as avoiding leading questions, response bias was avoided.

Because the data was collected using a single survey instrument, there was a chance that common method variance (CMV) would occur ( Podsakoff and Organ, 1986). Harmon’s single-factor test was carried out in the software IBM SPSS 26.0 to assess CMV. From the exploratory factor analysis of the data related to CEB, four components such as form/modality, scope, customers’ social media influence (CSMI) and the choice of channel, emerged which together define the CEB. Thus CEB became the formative second-order construct formed by the four reflective first-order constructs which were validated by literature review.

### Sample size

In terms of digital payments, Karnataka had the greatest adoption rate (26.64%), followed by Maharashtra (15.92%), and Delhi NCR (13%). Hence, Karnataka was picked as the geographical area based on this information. The tentative final sample size was calculated to be 382 customers using the formula:

$$N=\left(z^{2}*p*q.N_{U}\right)/\left(e^{2}\left(N_{U}-1\right)+z^{2}*p*q\right)$$

where p = Proportion of defectives in the universe (Based on the pilot study, a 2% defect is assumed); q = (1 – p); z = 1.96 (as per table of scores in a normal distribution within a selected range of z for a confidence level of 95%); e = Acceptable error (an error of 2% of the true value is assumed); N _U_ = Size of Universe.

After adding 10% to the calculated sample size to accommodate non-response errors, the target sample size was 420.

### Data analysis

The sample was measured for sample adequacy using the KMO test and Bartlett’s Test of Sphericity using IBM SPSS 26.0. A principal component analysis was conducted to ascertain patterns in data and to condense the variables to a more manageable level. Based on the criterion of Kaiser (1960), all factors with eigenvalues 1 or greater than 1 were retained. It was observed that KMO values for all constructs involved in this model were ≥ 0.5 ( Cerny & Kaizer, 1977) indicating that factor analysis is an appropriate method for further data analysis. Structural equation modelling (SEM) stating the path model and estimation of various quality parameters was analysed and reported using the Smart PLS 3.00 software, abiding by the latest guiding principles on Partial Least Squares ( Hair *et al*., 2013; Henseler *et al*., 2012). First, the measurement model was evaluated to check the reliability and validity of various constructs. Smart PLS 3.0 software was used to validate and analyze this hierarchical reflective-formative model of CEB through the disjoint two-stage approach. The relevance of the hypothesised associations between the constructs was then determined using the structural model. Alternatively, JASP which is an open-source PLS 3 software can be used to undertake SEM using lavaan R package based on a covariance-based approach.

## Results

Out of the 420 responses, it was found that 25 responses had incomplete responses and 15 other responses had a straight-lining pattern, hence 40 responses were discarded and 380 responses remained to be analysed ( Nayak Kini & Basri, 2021a). Almost 46% of respondents were found to use Google Pay, 17% each were found to use Phonepe and PayTM followed by Yono (SBI), Imobile (ICICI), BHIM and the others (fig 2). Almost 55% were males and 45% females and half of this sample population were in the age group of 25-40 years (millennials) earning anywhere between $1-$13,468 per year (1 US$ = Rs 74.25, 03 August 2021). On examining the sample, it is observed that 38% of the population is from urban, 46% from semi-urban (46%) areas, about 11% from metros and only about 3% from rural areas. The educational background of more than half of the sample (57%) was found to be postgraduates ( Table 1).

**Table 1.** Respondents’ demographic and FinTech app usage profile.

		Counts	Percentage (%)
**Gender**	Male	210	55
	Female	170	45
**Age**	Below 25	116	31
	25 – 40	188	49
	41 – 60	69	18
	60 +	7	2
**Educational qualification**	Undergraduate	23	6
	Graduate	91	24
	Post Graduate	215	57
	Doctorate	51	13
**Annual income**	Below 1 Lac	86	23
	1 Lac – 5 Lac	111	29
	5 Lac – 10 Lac	123	32
	Above 10 lac	60	16
**Financial app used**	Google Pay	175	46
	Phone Pe	66	17
	PayTM	63	17
	SBI Yono	29	8
	Imobile	14	4
	BHIM/UPI	12	3
	Others	20	5

### Measurement model

First, the lower-order constructs were taken into consideration and reliability and validity of the constructs were calculated for the reflective measurement models (refer to Table 2) For reliability, Cronbach’s alpha and composite reliability were checked and for validity, convergent validity and discriminant validity were assessed. Cronbach's α value for each construct is well above the threshold limit of α ≥ 0.70 ( Henson, 2001). Convergent validity was found to be acceptable as the average variance extracted (AVE) was greater than 0.5. Indicator reliability for each indicator was observed to be 0.70 or higher and was found acceptable ( Hulland, 1999). Discriminant validity was assessed by heterotrait-monotrait (HTMT) ratio of correlation ( Henseler *et al*., 2015), with values below the threshold of 0.9 acceptable. Multi-collinearity of each construct was checked where variance inflation factor (VIF) values were found to be below 5 hence they are moderately correlated and are not evidenced to create any issue. The assessment of the measurement model substantiated that all the construct measures are reliable and valid.

**Table 2.** Construct reliability, validity & multi-collinearity of survey items.

Construct	Indicators	CA	CR	AVE	OL	IR	VIF
Communal focus	CF1: I am going to speak up when the other fans of your brand community might be going to face a harmful situation.	0.828	0.897	0.745	0.868	0.753	2.040
	CF2: I complain when my brand community members see a harmful situation coming.				0.906	0.821	2.356
	CF3: I engage in a negative WOM when we are going to face a hurtful situation.				0.813	0.661	1.659
Customer advocacy	CA1: Generally, I would recommend my FinTech apps to my friends and family.	0.834	0.889	0.668	0.816	0.666	1.815
	CA2: I promote the brand because of the monetary referral benefits provided by the brand.				0.771	0.594	1.615
	CA3: When I hear people speaking badly about my app I try to defend it.				0.817	0.667	1.775
	CA4: I insist my family and friends use my FinTech app.				0.864	0.746	2.128
Customer engagement behaviour	CSMI: Customers’ Social Media Influence.	0.891	0.925	0.754			2.382
	CEB_1: I feel an emotional link with my app/company.				0.836	0.781	2.033
	CEB_2: I actively discuss this app with other customers on social media.				0.893	0.797	2.920
	CEB_3: I seek advice from other customers on how to solve the problems.				0.856	0.733	2.398
	CEB_4: I love talking about the benefits and positive app experiences with other customers on social media.				0.886	0.785	2.920
	FM Form/Modality:	0.894	0.923	0.705			2.585
	CEB _5: I would organize a public action against the firm in the case of a dispute.				0.826	0.682	2.187
	CEB _6: I tend to express my experiences through blogs.				0.758	0.574	1.663
	CEB _7: I actively participate in firm-organized charity events, donating money and time.				0.886	0.785	3.044
	CEB _8: I generally donate through charity events but do not have the time to participate in them.				0.888	0.788	3.288
	CEB _9: I tend to complain about the app/firm on social media forums.				0.834	0.695	2.470
	Choice of Channel *:* Preference for communication channel while dealing with other customers and company.	0.867	0.900	0.602			1.350
	CEB _10: with other customers in-person.				0.755	0.571	1.829
	CEB _11: with other customers via the Internet (social media or website).				0.783	0.614	2.551
	CEB _12: with other customers via phone, mail, or e-mail.				0.834	0.696	2.896
	CEB _13: with the company in-person customer to firm.				0.750	0.563	1.756
	CEB _14: with the company via the Internet (social-media or website).				0.790	0.624	2.517
	CEB _15: with the company via phone/mail/e-mail.				0.736	0.542	2.286
	*Scope*	0.926	0.947	0.819			2.796
	CEB _16: My product-related expressions and actions help my company.				0.847	0.718	2.180
	CEB _17: I provide feedback about my app experiences to the firm.				0.917	0.840	3.672
	CEB ^_^18: I provide suggestions for improving the performance of the app.				0.939	0.883	4.229
	CEB ^_^19: I provide feedback/suggestions for developing new service offerings for my app.				0.913	0.834	4.131
Emotions	Happiness: Unhappy/Happy	0.876	0.915	0.728	0.874	0.767	2.449
	Contentment: Discontented/Contented				0.836	0.699	2.070
	Pleasure: Displeased/Pleased				0.892	0.796	2.657
	Frustration: Enjoyable/Frustrating				0.81	0.656	1.843
Moral identity	MI1: I want to warn others of bad financial applications.	0.828	0.876	0.642	0.694	0.482	2.325
	MI2: I want to save others from having the same negative experiences as me.				0.702	0.493	2.412
	MI3: I want to help others with my own positive experiences.				0.911	0.830	3.061
	MI4: I want to allow others to install/use the right financial application.				0.882	0.778	2.898
Perceived benefits	PB1: I manage to doFinTech transactions in the least amount of time.	0.909	0.930	0.687	0.772	0.596	2.019
	PB2: It has useful features.				0.857	0.734	2.753
	PB3: This financial application gives me better deals.				0.847	0.717	2.486
	PB4: The app has exclusive time-bound offers.				0.831	0.691	2.363
	PB5: While shopping through the app, I find what I'm looking for quickly.				0.843	0.711	2.654
	PB6: I expend little effort to do transactions through FinTech compared to other channels.				0.821	0.674	2.380
Perceived cost	PC1: App installation cost is not very high	0.642	0.800	0.574	0.68	0.462	1.330
	PC2: Transaction processing cost with FinTech applications is high.				0.715	0.511	1.193
	PC3: These applications help me save money.				0.856	0.733	1.291
Self-concept	SC1: I identify with what my company or app stands for.	0.879	0.912	0.674	0.798	0.637	2.236
	SC2: I feel a sense of belonging concerning my company.				0.845	0.714	2.524
	SC3: I bring up things I have seen on this app in conversations with other people.				0.794	0.630	1.821
	SC4: When I talk about this brand, I usually say ‘we’ rather than they.				0.842	0.709	2.539
	SC5: This brand’s successes are my successes.				0.826	0.682	2.462

CA = cronbach alpha; CR = composite reliability; AVE = average variance extracted; OL = outer loadings (standardised); IR = indicator reliability; CEB = customer eengagement behaviour; VIF = variance inflation factor; CF = communal focus; MI = Moral identity; PB = perceived benefits; PC = perceived cost; CA = customer advocacy; SC = self-concept.

The latent scores of the four lower-order constructs of CEB were added to the data set before running stage two. Here the four latent variable constructs acted as the indicators of the higher-order construct CEB. The measurement model of the formative higher-order construct (HOC) CEB was validated by running the bootstrapping procedure with 5000 samples. The outer weights were checked for significance, here the outer weight of FM (form/modality) was < 0.5 and that of scope, CSMI and channel were below 0.5, hence the outer loading was then checked which was > 0.5 and was thus found to be significant.

### Structural model assessment

The structural model was assessed using the principal measure of assessment which is the coefficient of determination R ^2^ ( Henseler *et al*., 2012). The high R ^2^ value of 0.465 for customer engagement behaviour and 0.495 for customer advocacy signifies that almost 46.5% of the variance in CEB is explained by its antecedents which are emotions, self-concept, communal focus, perceived cost and benefits. Also, half of the variance in customer advocacy is explained by CEB. The high R ^2^ values thus substantiate the model's predictive validity. The model fit indices were checked. SRMR value is 0.068 which is < 0.10 or 0.08 (Hu and Bentler, 1999) and NFI (normed fit index) of 0.668 represents an acceptable fit. RMS_theta is 0.104 which is well below the threshold value of 0.12.

### Direct effects

The direct and indirect effects of various constructs were analyzed. The path-coefficient ‘β’ of a path, its t statistics, p-value, effect size f ^2^ and whether hypotheses on direct paths are supported or not are outlined in Table 3. In terms of variables having the highest influence on CEB suggested by path-coefficient, self-concept has the highest influence on CEB (β = 0.366) followed by communal focus (β = 0.184), perceived benefits (β = 0.118) and emotions (β = 0.110). The f ^2^ values signify the effect sizes of the path. The path self-concept> CEB has the highest f ^2^ value 0.145 followed by communal focus (f ^2^ = 0.095), perceived benefits (f ^2^ = 0.023), suggesting a small effect and emotions with an f ^2^ value of 0.019 suggesting that there is no effect of emotions on CEB’s R ^2^.

**Table 3.** Direct effects and effect sizes (f ^2^) of customer predispositions and engagement on advocacy behaviours.

Hypothesised path relationships	β	t	p	f ^2^	Hypothesis
H1: CEB -> customer advocacy	0.303	5.032	0.000	0.098	Supported
H2: Emotions -> CEB	0.110	2.225	0.026	0.019	Supported
H3: Emotions -> customer advocacy	0.198	4.389	0.000	0.065	Supported
H4: Self-concept -> CEB	0.366	6.625	0.000	0.145	Supported
H5: Self-concept -> customer advocacy	0.101	1.719	0.086	0.010	Not supported
H6: Moral identity -> CEB	0.007	0.149	0.881	0.000	Not supported
H7: Moral identity -> customer advocacy	−0.014	0.265	0.791	0.000	Not supported
H8: Communal focus -> CEB	0.184	3.225	0.001	0.040	Supported
H9: Communal focus -> customer advocacy	0.279	5.264	0.000	0.095	Supported
H10: Perceived cost -> CEB	0.064	1.051	0.294	0.005	Not supported
H11: Perceived benefits -> CEB	0.118	1.967	0.049	0.023	Supported

CEB = customer engagement behaviour.

CEB, communal focus and emotions are found to have a significant effect on customer advocacy as suggested by path coefficients 0.303, 0.279 and 0.198 respectively and the effect sizes f ^2^ values of 0.098, 0.095 and 0.065 respectively. Again, based on the t and p-value, moral identity has an insignificant effect both on CEB and customer advocacy whereas perceived cost has an insignificant effect on CEB.

### Indirect effects

The mediator CEB necessitates the need for checking the significance of indirect effects for the model. The indirect effect of self-concept on customer advocacy is the highest, followed by the communal focus and perceived benefits as suggested by the t and the p values as well as the path coefficients in Table 4. However, there is no significant indirect influence of perceived cost, moral identity or emotions on customer advocacy.

**Table 4.** Indirect effects of customer predispositions on advocacy behaviours mediator being customer engagement behaviour (CEB).

Path	Path coefficients	t	p	Significance
Perceived cost -> CEB -> customer advocacy	0.019	1.020	0.308	Not significant
Moral identity -> CEB -> customer advocacy	0.002	0.115	0.908	Not significant
communal focus -> CEB -> customer advocacy	0.056	2.506	0.012	Significant
Self-concept -> CEB -> customer advocacy	0.111	4.115	0.000	Significant
Emotions -> CEB -> customer advocacy	0.033	1.815	0.070	Not significant
Perceived benefits -> CEB -> customer advocacy	0.052	2.230	0.026	Significant

### Mediation effects

Customer engagement behaviour fully mediates the relationship between self-concept and customer advocacy (CA) as the direct relation between them is insignificant whereas the indirect relation is significant. CEB partially mediates the path between perceived benefits and CA with a VAF (variance accounted for) = 0.306 (30.6%), the path communal focus and CA with a VAF = 0.166 (16.6%). CEB does not mediate the other paths such as emotions -> CA, perceived cost -> CA or moral identity -> customer advocacy.

VAF (Variance Accounted For) is calculated using the formula

VAF=Direct Effect/Total Effect(Direct+Indirect Effect)

## Discussion

In the FinTech industry, the extensive usage of innovative technology has drastically reduced the direct face-to-face customer communications requiring experiential elements to be cherished. Our study result empirically suggests that CEBs displayed by e-WOM (e-word of mouth) behaviour are predominantly experiential and result in non-transactional outcomes such as advocacy. Study results show self-concept has a direct effect on CEB supporting the Levy and Hino (2016) study which encourages social media conversations based on a sense of brand belongingness. Self-concept also shows an indirect effect on advocacy that is fully mediated by CEB. This means that customers with a positive self-concept associated with the product tend to advocate the product only if they are engaged positively. Thus, managers need to focus on identifying and nurturing the self-concept of their customers where customers identify themselves with the brand, for them to engage. This can be achieved by having individual 'thought leaders' whose opinions on products are followed by the customers to make buying or usage decisions. Customers' product benefit perceptions can engage customers positively but cost perceptions of the FinTech transaction do not inspire them to propagate any e-WOM to their brand community.

The study results show that the emotional dimension of engagement has a direct positive effect on both engagement and advocacy (e-WOM). If emotions are positive, customers tend to experience a positive CEB or recommend the product to other customers in their social media community thus being favourable to the company, validating the previous studies by Garg (2005) and Levy and Hino (2016). If emotions are negative customers end up with negatively valenced CEB making customers less likely to recommend products. This also confirms previous studies on negative emotions linked to disengagement ( van Doorn *et al.*, 2010). Study results suggest customers' propensity to help others does not result in e-WOM or a referral campaign refuting previous studies by Sundaram (1998) and van Doorn *et al.* (2010).

Our study propositions companies to design and build the products and applications to address customer needs. Companies now need to invest in creating unique engaging experiences accommodating customers' perceptions on product benefits, their inherent communal focus, self-concept and emotional needs in mind for them to engage and promote the brand to others turning them into advocates. FinTech firms need to primarily have platforms and social media channels that nurtures C2C interactions, reviews, and testimonials favourable for the FinTech brands and companies. The companies can inspire improved perceptions on product benefits by implementing necessary mechanisms that make the transactions safer, faster, smoother, and more convenient. In addition, FinTech companies should incorporate innovative features, deals and offers customised to the past app user behaviour and the market needs. Moreover, it becomes essential not to hurt the communal feelings of the product community giving way to any unfavourable CEB such as a negative WOM. This implies that brands need to be focused and sensitive to the needs of community groups, which otherwise can turn into a negative engagement affecting a larger customer base.

This empirical study authenticates the role of CEB as a mediator fully mediating the relationship path self-concept -> customer advocacy and partially mediating the relationship paths perceived benefits -> customer advocacy and communal focus -> customer advocacy. Correspondingly, it assists practitioners to devise suitable and personalised segmentation or promotional strategies for various marketing campaigns.

### Practical implications

This paper provides directions for marketing managers, e-marketers, technologists, academicians, and practitioners of engagement marketing in the FinTech industry. It guides the managers on how best to engage customers through self-concept, emotional connect and by suitably managing customer predispositions so that they display positive advocacy behaviours (reviews/testimonials). These insights may help the firms to enhance personalization and digital engagement in real-time and improve customer experience by responding to customer needs through co-created offerings. The knowledge of the factors which motivate customers to engage or disengage and prevailing customers’ level of engagement/disengagement may help in designing a robust classification and segmentation strategy. Hence, this study principally assists FinTech managers on the ways to devise and implement the right marketing strategy based on various customer needs leading to continued advocacy behaviours.

### Research limitations and future research agenda

The data collected for this quantitative study was from the financial app users of the southern region of India alone, hence it may limit the external validity of its results. The respondents used an online form to share their responses during lockdown causing a biased response due to COVID-19. Also, since the study was purely quantitative, it would have failed to capture other dynamic and subjective ideas of engagement behaviour in this ever-evolving, emerging FinTech industry which would have been possible with a mixed-method approach involving both quantitative and qualitative techniques.

The study could be simulated for other service industries such as e-commerce, healthcare, education, tourism, media and entertainment which dwell on non-transactional behaviours. As observed, there are different types of CEBs such as e-WOM, referrals, reviews, recommendations, testimonials, and blogging, with CEBs being specific to the particular industry. Correspondingly, paths of customer predispositions>CEB>customer advocacy in this model for the FinTech industry can be studied with certain demographic variables as the moderators. Similar to customer predispositions influencing engagement and advocacy behaviours, a positive recommendation and e-WOM could trigger positive emotions, communal focus, self-concept or cost/benefit perceptions and make the customer engage with the brand. This circular nature of relationships is a promising new area for future research. Studies with various population groups and cultures, for firm-based, and context-based factors could be built-in. Apart from customer-based factors, extrinsic system level factors handling security and safety features influencing CEB and customer advocacy are also a great possibility. A qualitative approach to understanding CEBs for a specific industry or a population group may prove insightful. A longitudinal study of CEBs may be desirable as the customers’ perceptions keep evolving with time as new products and technologies emerge. Moreover, future studies could also explore these C2C interactions in different product or market settings where the nature of the precursors to customer advocacy may be altered.

## Conclusion

It is observed that the emerging financial services companies are continuing to digitise and technologically disrupt. Especially, during the COVID-19 pandemic, as the adoption of technology-enabled contactless financial services rises, it becomes vital to meet the increasing customer expectations by assisting them in making correct choices and accomplishing customer goals. Apart from that, firms need to individualise, engage, and personalise the offerings to stand out in the ever-growing competition. The study results indicate that a highly pre-dispositional customer possessing a high self-concept reciprocate positive e-WOM only when they are engaged with the financial application created through online influencer strategies. The positive CEBs get formed through self-concept, communal focus, emotions and perceived benefits related to the product helping to build advocacy behaviours. Results also indicate that emotions alone can directly influence advocacy for the FinTech industry. Thus, the emerging and competitive FinTech industry survives only if the emotionally connected customers, whose self-concept, communal focus and perceptions on product benefits are aligned with that of the companies demonstrating long-term brand engagement advocacy behaviours.

## Data availability

### Underlying data

Mendeley Data: Raw data CSV processed for use in smartPLS-Fintech India-customer predispositions-engagement-advocacy behaviour. https://doi.org/10.17632/5j6csksgb4.5 ( Nayak Kini & Basri, 2021a).

### Extended data

Mendeley Data: Survey form - Fintech India Customer predispositions-Engagement-Advocacy. https://doi.org/10.17632/dfxjk69h8m.4 ( Nayak Kini & Basri, 2021b).

Mendeley Data: Information sheet and written consent form - FinTech India. https://doi.org/10.17632/njz464h8f8.1 ( Nayak Kini & Basri, 2021c).

Data are available under the terms of the Creative Commons Attribution 4.0 International license (CC-BY 4.0).
