# Supplementary material for: An empirical examination of customer advocacy influenced by engagement behaviour and predispositions of FinTech customers in India
Source: F1000Res. 2022 May 23;11:27. Originally published 2022 Jan 11. [Version 2] doi: 10.12688/f1000research.74928.2 (PMC9135099; doi:10.12688/f1000research.74928.2)
Supplement: Peer review history

# Peer review: Reviewer response for version 2

**DOI:** 10.5256/f1000research.134231.r138622

**Published:** 2022-05-25

**Authors:** Prashant Kumar

**Affiliations:** 1 School of Management, Asian Institute of Technology, Pathumthani, Thailand

Thank you for addressing my comments and revising the manuscript. I believe all of the comments are appropriately addressed and the manuscript in its current form is ready for indexing.

Is the work clearly and accurately presented and does it cite the current literature?

Yes

If applicable, is the statistical analysis and its interpretation appropriate?

Yes

Are all the source data underlying the results available to ensure full reproducibility?

Yes

Is the study design appropriate and is the work technically sound?

Yes

Are the conclusions drawn adequately supported by the results?

Yes

Are sufficient details of methods and analysis provided to allow replication by others?

Yes

Reviewer Expertise:

NA

I confirm that I have read this submission and believe that I have an appropriate level of expertise to confirm that it is of an acceptable scientific standard.

# Peer review: Reviewer response for version 2

**DOI:** 10.5256/f1000research.134231.r138623

**Published:** 2022-05-23

**Authors:** Naveen Kumar K

**Affiliations:** 1 National Institute of Bank Management, Pune, Maharashtra, India

The revised article makes a perfect reading and much more clarity. Congratulations on the efforts.

Is the work clearly and accurately presented and does it cite the current literature?

Yes

If applicable, is the statistical analysis and its interpretation appropriate?

Yes

Are all the source data underlying the results available to ensure full reproducibility?

Yes

Is the study design appropriate and is the work technically sound?

Yes

Are the conclusions drawn adequately supported by the results?

Yes

Are sufficient details of methods and analysis provided to allow replication by others?

Yes

Reviewer Expertise:

NA

I confirm that I have read this submission and believe that I have an appropriate level of expertise to confirm that it is of an acceptable scientific standard.

# Peer review: Reviewer response for version 1

**DOI:** 10.5256/f1000research.78732.r135480

**Published:** 2022-05-06

**Authors:** Prashant Kumar

**Affiliations:** 1 School of Management, Asian Institute of Technology, Pathumthani, Thailand

The paper is well written - it follows the structure of a standard journal article, starting with introduction, literature review, method, results and discussion. It explains the research gaps and discusses at the end how the study addressed the gaps. The analysis is extensive and results are well-presented. 

 Please address following comments:

- Write hypotheses in cause and effect format e.g. emotions have a positive effect on CEB.
- In measurement scales, write how many items each scale contained.
- In sample size, include how many respondents were contacted and how many responded and thus what was the response rate.

Thank you.

Is the work clearly and accurately presented and does it cite the current literature?

Yes

If applicable, is the statistical analysis and its interpretation appropriate?

Yes

Are all the source data underlying the results available to ensure full reproducibility?

Yes

Is the study design appropriate and is the work technically sound?

Yes

Are the conclusions drawn adequately supported by the results?

Yes

Are sufficient details of methods and analysis provided to allow replication by others?

Yes

Reviewer Expertise:

NA

I confirm that I have read this submission and believe that I have an appropriate level of expertise to confirm that it is of an acceptable scientific standard, however I have significant reservations, as outlined above.

# Response

**Published:** 2022-05-19

**Authors:** Archana Nayak Kini

**Affiliations:** Manipal Institute of Management, India

Thank you for your review comments. Please find my point by point responses below.

**Q1.** **Write hypotheses in cause and effect format e.g. emotions have a positive effect on CEB.**

**Answer:** This has been revised as per suggestions and has been incorporated in the version 2 of the paper

 H1: There is a relationship between customer engagement behaviour (CEB) and customer advocacy.

 Changed to

**H1:** **Customer engagement behaviour positively impacts customer advocacy**.         

 H2: There is a relationship between emotions and CEB.

 Changed to

**H2: Emotions have a positive effect on CEB**

 H3: There is a relationship between emotions and customer advocacy.

 Changed to

**H3: Emotions have a positive influence on customer advocacy**.

 H4: There is a relationship between self-concept and CEB

 Changed to

**H4: Self-concept impacts CEB positively.**

 H5: There is a relationship between self-concept and customer advocacy.

 Changed to

**H5: Self-concept impacts customer advocacy positively.**

 H6: There is a relationship between moral identity and CEB.

 Changed to

**H6: Moral Identity influences CEB in a positive manner.**

 H7: There is a relationship between moral identity and customer advocacy.

 Changed to

**H7: Moral Identity inspires customer advocacy in a positive manner.**

 H8: There is a relationship between communal focus and CEB.

 Changed to

**H8: Presence of communal focus is positively associated with CEB.**

 H9: There is a relationship between communal focus and customer advocacy.

 Changed to

**H9: Customer advocacy responds positively to moral identity.**

 H10: There is a relationship between perceived cost and CEB.

 Changed to

**H10: CEB is positively influenced by perceived cost.**

 H11: There is a relationship between perceived benefits and CEB.

 Changed to

**H11: CEB is positively motivated by perceived benefits.**

**Q2**: **In measurement scales, write how many items each scale containe**d.

**Answer**: The survey questionnaire used a 5-point Likert scale adapted from several researchers such as Levy and Hino (2016) to capture customers’ overall perceptions about **self-concept having 5 items**, van Doorn *et al.* (2010) for communal focus with 3 items, Hennig-Thurau *et al*. (2004) for **moral identity having 4 items**, Hayashi and Bradford (2014) for **perceived cost having 3 items, perceived benefits having 6 items** and Moliner *et al*. (2018), Kumar and Pansari (2016), for **CEBs having 18 items**, and Moliner *et al*. (2018) and Han *et al*. (2008) for **advocacy concerning the specific apps they were using and having 5 items.** For **emotions**, a 7-point Likert scale **having 4 items** which was adapted from Wong (2004) was used. The survey can be found as *Extended data* ( Nayak Kini & Basri, 2021b).

The number of items have been updated in the version 2 of the paper as well.

**Q3. In sample size, include how many respondents were contacted and how many responded and thus what was the response rate.**

**Answer:** We contacted 450 respondents and 420 of them responded with a response to complete the survey. Thus the response rate was (420/450) *100 = 93%

 Response rate has been updated in the Methods>Data Collection section of the paper as well.

# Peer review: Reviewer response for version 1

**DOI:** 10.5256/f1000research.78732.r119411

**Published:** 2022-01-27

**Authors:** Naveen Kumar K

**Affiliations:** 1 National Institute of Bank Management, Pune, Maharashtra, India

While the number of FinTechs in India is on the rise to tap business opportunities emerging from the Covid-19 pandemic, the task before them is how to become friendlier to customers. In this regard, the paper aims at empirically testing the influence of customer-based antecedents such as emotions, moral identity, self-respect, customer focus, perceived cost, and product benefits for marketing. The paper considers major FinTechs operating in Karnataka State and concludes based on analysis of responses to the structured questionnaire from 380 customers from the same region. The paper employs Structural Equation Modeling using Smart Partial Least Square 3.0 to test the hypotheses. Results discussed in the paper are quite interesting, logically convincing, and practically oriented. Based on the study results, antecedents statistically found significant for engagement of customers include product benefits perception, self-respect, emotional contact, and customer predispositions. 

**Q1.  Is the work clearly and accurately presented and does it cite the current literature?**

 Yes, the research findings are clearly and accurately discussed in the paper. The author deserves appreciation in mentioning a large number of earlier studies on the related aspects of the subject and setting the hypotheses in continuation of findings of the same. Among the customer-based antecedents mentioned in the paper, it is suggested to examine the relationship between safety & security on one hand and customer advocacy on the other, based on the responses received from the respondents. This suggestion is made, taking into account the increasing number of frauds in online financial dealings in India. To elaborate, the share of suspected digital fraud attempts against financial services businesses increased by 89 percent during January-May, 2021.Therefore, the author is suggested to mention the importance of safety in FinTech dealings in the paper, which would be one of the considerations of customers. To know more about the current concerns relating to frauds in FinTech business, the author may go through these references: ^1^, ^2^, ^3^, ^4^

**Q2.  Is the study design appropriate and is the work technically sound?**

 Yes, the study design seems to be appropriate and the work technology is also sound. One more consideration should be made regarding the sample design. Currently, digital payment is limited largely to the population in urban and metro areas. Therefore, there is a felt need to increase the penetration of FinTech to the vast sections of the population which is unbanked and lacks a smartphone. Towards this end, the author may discuss the location of 380 respondents into Metro and Urban and Semi-urban and Rural and analyse responses accordingly. Quite likely, expectations of the customers from FinTech shall vary from one area to another.

**Q3.  Are sufficient details of methods and analysis provided to allow replication by others?**

 Yes. But, it is suggested to mention the following aspects in the paper::

1. Major contents of the questionnaire
2. Regarding the selection of FinTechs, the Payment Banks are not considered. But, this bank has now more than 5 crores customers. Therefore, the reason for not including the same should be stated.
3. Details of the profession of the respondents in the sample should be shared in the paper, since the use of FinTech varies from fixed income group users to business income group users.

** **


**Q4. If applicable, is the statistical analysis and its interpretation appropriate?**

 Statistical analysis and interpretation are very much appropriate and impressive. Statistical tools/tests are also in line with the earlier studies referred to in the paper. Two suggestions are made relating to analysis and interpretation:

- The significance of each statistical tool/test stated in the paper shold be explained in simple terms for the benefit of the readers and,
- The paper should state whether it attempts to make any change in the statistical tools as employed in the earlier studies.

 

**Q5. Are all the source data underlying the results available to ensure full reproducibility?**

 Yes. Future work may be looked into the interaction between primary and secondary analysis.

**Q6.  Are the conclusions drawn adequately supported by the results?**

****

 The author deserves full appreciation for carrying out the extensive statistical analysis to test the hypotheses. The results of the analysis are almost in line with the earlier studies. Findings of the study make a significant contribution to the existing fund of knowledge.

Is the work clearly and accurately presented and does it cite the current literature?

Yes

If applicable, is the statistical analysis and its interpretation appropriate?

Yes

Are all the source data underlying the results available to ensure full reproducibility?

Yes

Is the study design appropriate and is the work technically sound?

Yes

Are the conclusions drawn adequately supported by the results?

Yes

Are sufficient details of methods and analysis provided to allow replication by others?

Yes

Reviewer Expertise:

NA

I confirm that I have read this submission and believe that I have an appropriate level of expertise to confirm that it is of an acceptable scientific standard.

## References

[1] : Committee on Comprehensive Financial Services for Small Businesses and Low Income Households. *Reserve Bank of India* .2013; Reference source

[2] : FinTechs and Virtual Currency. *FinTech Conference 2017* .2017; Reference source

[3] : Responsible Digital Innovation. *Global Fintech Festival* .2021; Reference source

[4] Consultation Paper on Peer to Peer Lending. *Reserve Bank of India* .2016; Reference source

# Response

**Published:** 2022-05-19

**Authors:** Archana Nayak Kini

**Affiliations:** Manipal Institute of Management, India

**Q1. Is the work clearly and accurately presented and does it cite the current literature?**

 Yes, the research findings are clearly and accurately discussed in the paper. The author deserves appreciation in mentioning a large number of earlier studies on the related aspects of the subject and setting the hypotheses in continuation of findings of the same. Among the customer-based antecedents mentioned in the paper, it is suggested to examine the relationship between safety & security on one hand and customer advocacy on the other, based on the responses received from the respondents. This suggestion is made, taking into account the increasing number of frauds in online financial dealings in India.

**Q1 Answer:**

 The purview of our study is exclusively for a sample of customers who have adopted online payments using apps such as GooglePay, PhonePe, BHIM which is one of the broader offerings under FinTech. Other offerings include peer to peer lending, virtual currency such as cryptos and NFTs (non-fungible tokens), insurance as mentioned in the articles 1, 2, 3, 4 related to the online frauds in the FinTech industry. The security and fraud detection related to the online payments are already being managed using face recognition, AI (Artificial Intelligence) and ML (Machine Learning) ( Tekkali & Vijaya, 2021). The Fraud Detection System allows one to detect illegal actions and malicious behaviours and efforts before they cause major damage to the firm and customers ( Gupta & Jhamb, 2020; Samet, 2013). Online payments’ system design, architecture, authentication mechanisms and algorithms being system level factors which manage the security and privacy requirements are extrinsic in nature and are not considered for this study. This exclusion has been included as a research limitation of the study.

 The main focus thus is on the intrinsic factors of the customers such as emotions, their perceptions about product cost & benefits, their moral identity, self-concept and communal focus. These factors were identified from literature as they were predominantly influencing customer engagement behaviours in the tech-enabled financial services ( Van Doorn et al., 2010. CEB (Customer Engagement Behaviour) is a behavioural construct that is primarily non-transactional in nature and goes beyond purchases and financial transactions. It is proven that emotional and behavioural factors, rather than a simply transactional strategy, influence a bank's relationship with consumers, resulting in bank advocacy ( Levy & Hino, 2016; Bhat & Darzi, 2016).

**Q2.  Is the study design appropriate and is the work technically sound?**

 Yes, the study design seems to be appropriate and the work technology is also sound. One more consideration should be made regarding the sample design. Currently, digital payment is limited largely to the population in urban and metro areas. Therefore, there is a felt need to increase the penetration of FinTech to the vast sections of the population which is unbanked and lacks a smartphone. Towards this end, the author may discuss the location of 380 respondents into Metro and Urban and Semi-urban and Rural and analyse responses accordingly. Quite likely, expectations of the customers from FinTech shall vary from one area to another.

**Q2 Answer:**

 On examining the sample, it is observed that 38% of the population is from urban, 46% from semi-urban areas, about 11% from metros and only about 3% from rural areas. As the majority of the sample population i.e. 84% is in semi-urban and urban, data analysis of this paper also demonstrates the expectations and behaviours of these customers. Therefore, future studies can focus on rural areas.

The location of the sample is incorporated in the ‘Results’ section of the paper.

**Q3.  Are sufficient details of methods and analysis provided to allow replication by others?**

 Yes. But, it is suggested to mention the following aspects in the paper.

1. Major contents of the questionnaire **-Q3 >1 >Answer**: The survey questionnaire used a 5-point Likert scale adapted tools from several researchers such as Levy and Hino (2016) to capture customers’ overall perceptions about self-concept having 5 items, van Doorn et al. (2010) for communal focus with 3 items, Hennig-Thurau et al. (2004) for moral identity having 4 items, Hayashi and Bradford (2014) for perceived cost having 3 items, perceived benefits having 6 items and Moliner et al. (2018), Kumar and Pansari (2016), for CEBs having 18 items, and Moliner et al. (2018) and Han et al. (2008) for advocacy concerning the specific apps they were using and having 5 items. For emotions, a 7-point Likert scale having 4 items which was adapted from Wong (2004) was used. The survey questionnaire can be found as ‘Extended data’ ( Nayak Kini & Basri, 2021b).The number of items in the scale for each construct has been incorporated in the paper.
2. Regarding the selection of FinTechs, the Payment Banks are not considered. But, this bank has now more than 5 crores customers. Therefore, the reason for not including the same should be stated. **Q3> 2> Answer:** Payment banks (PB) are a model of banks introduced to achieve financial inclusion in a cashless economy back in 2015. They accept cash deposits from customers which is stored in digital wallets and used for payments and remittances, but they do not offer loans and credits unlike traditional banks. The services targeted migrant labour, low income households, farmers, small businesses and other users in mind. Unfortunately, lower adoption rate was observed in lower income groups due to lack of awareness, low level of trust and lesser perceived need for these services (Pramani & Iyer, 2022). The PBs have continued to incur losses which have risen through 2017 till 2020 with the net consolidated loss as on March 2020 to be at INR 833 crores (RBI, 2020). 
  On the other hand, based on the various research reports, FinTech adoption rate has been constantly rising and it was the highest for India surpassing China (Ernst & Young, 2021). Indian states such as Karnataka saw the highest rate of 26.64%, followed by Maharashtra (15.92%), and Delhi NCR (13%) (Razorpay, 2020). In terms of digital payments, post COVID-19 disease outbreak, PhonePe, Googlepay, Policybazaar and other similar financial apps were extensively adopted and used contributing to an exponential trend unlike payment banks (RBI 2021). Hence the purview of the study included only these app-oriented behaviours which are non-transactional in nature.
3. Details of the profession of the respondents in the sample should be shared in the paper, since the use of FinTech varies from fixed income group users to business income group users.

**Answer to Q3>3**: The study considered majorly the fixed income group individuals with only about 7% of the sample population being business income users. The moderation analysis considering occupation as the moderator did not show any differences between these two groups of users. We have excluded the write-up on this analysis for brevity of the paper.

**Q4. If applicable, is the statistical analysis and its interpretation appropriate?**

 Statistical analysis and interpretation are very much appropriate and impressive. Statistical tools/tests are also in line with the earlier studies referred to in the paper. Two suggestions are made relating to analysis and interpretation:

- The significance of each statistical tool/test stated in the paper should be explained in simple terms for the benefit of the readers and,
- The paper should state whether it attempts to make any change in the statistical tools as employed in the earlier studies.
** **

**Q4> 1> Answer : **The sample was measured for sample adequacy using the KMO test and Bartlett’s Test of Sphericity using IBM SPSS 26.0. Structural equation modelling (SEM) stating the path model and estimation of various quality parameters was analysed and reported using the Smart PLS 3.00 software, abiding by the latest guiding principles on Partial Least Squares ( Hair *et al*., 2013; Henseler *et al*., 2012).

 First, the measurement model was evaluated to check the reliability using cronbach’s alpha and composite reliability. Cronbach's α value for each construct is well above the threshold limit of α ≥ 0.70 ( Henson, 2001). Indicator reliability for each indicator was observed to be 0.70 or higher and was found acceptable ( Hulland, 1999).

 For validity, convergent validity and discriminant validity were assessed. Convergent validity was found to be acceptable as the average variance extracted (AVE) was greater than 0.5. Indicator reliability for each indicator was observed to be 0.70 or higher and was found acceptable ( Hulland, 1999). Discriminant validity was assessed by heterotrait-monotrait (HTMT) ratio of correlation ( Henseler *et al*., 2015), with values below the threshold of 0.9 acceptable.

 Multi-collinearity of each construct was checked where variance inflation factor (VIF) values were found to be below 5 hence they are moderately correlated and are not evidenced to create any issue.

 The structural model was assessed using the principal measure of assessment which is the coefficient of determination R ^2^ ( Henseler *et al*., 2012). The high R ^2^ value of 0.465 for customer engagement behaviour and 0.495 for customer advocacy signifies that almost 46.5% of the variance in CEB is explained by its antecedents which are emotions, self-concept, communal focus, perceived cost and benefits.The high R ^2^ values substantiate the model's predictive validity. 

 The model fit indices were checked. SRMR value is 0.068 which is < 0.10 or 0.08 (Hu and Bentler, 1999) and NFI (normed fit index) of 0.668 represents an acceptable fit. RMS_theta is 0.104 which is well below the threshold value of 0.12.

 The hypotheses were tested by analysing the direct and indirect effects. The significance of the path was tested using the path co-efficient, p-value and t stats of the paths in the model. Similarly, the mediation effects and the level of partial mediation was measured using the VAF (Variance accounted for) value which is the amount of direct effect divided by the amount of the total effect (direct effect + indirect effect). The formula for VAF has been updated in the version 2 of the paper.

​​​​​​​

**Q4> 2> Answer: **The study does not attempt to make any change in the statistical tool but adapts the scales for different constructs from several researchers. 

**Q5. Are all the source data underlying the results available to ensure full reproducibility?**

 Yes. Future work may be looked into the interaction between primary and secondary analysis.

**Answer to Q5**: Sure, that can be one of the potential future studies.

**Q6.  Are the conclusions drawn adequately supported by the results?**

 The author deserves full appreciation for carrying out the extensive statistical analysis to test the hypotheses. The results of the analysis are almost in line with the earlier studies. The findings of the study make a significant contribution to the existing fund of knowledge.

**References**

 Tekkali, C. G., & Vijaya, J. (2021, August). A Survey: Methodologies used for Fraud Detection in Digital Transactions. In *2021 Second International Conference on Electronics and Sustainable Communication Systems (ICESC)* (pp. 1758-1765). IEEE.

 Samet, O. (2013). *Introduction to online payments risk management*. " O'Reilly Media, Inc.".

 Gupta, N., & Jhamb, D. (2020). How India can develop its payments fraud prevention model: A study of emerging best practices. *Journal of Payments Strategy & Systems*, *14*(3), 237-255
